# Multifunctional Effects of Mangosteen Pericarp on Cognition in C57BL/6J and Triple Transgenic Alzheimer's Mice

**DOI:** 10.1155/2014/813672

**Published:** 2014-12-01

**Authors:** Hei-Jen Huang, Wei-Lin Chen, Rong-Hong Hsieh, Hsiu Mei Hsieh-Li

**Affiliations:** ^1^Department of Nursing, Mackay Junior College of Medicine, Nursing and Management, Taipei 112, Taiwan; ^2^Department of Life Science, National Taiwan Normal University, Taipei 116, Taiwan; ^3^School of Nutrition and Health Sciences, Taipei Medical University, Taipei 110, Taiwan

## Abstract

Mangosteen- (*Garcinia mangostana*-) based nutraceutical compounds have long been reported to possess multiple health-promoting properties. The current study investigated whether mangosteen pericarp (MP) could attenuate cognitive dysfunction. First, we found that treatment with MP significantly reduced the cell death and increased the brain-derived neurotrophic factor (BDNF) level in an organotypic hippocampal slice culture (OHSC). We then investigated the effects of age and MP diet on the cognitive function of male C57BL/6J (B6) mice. After 8-month dietary supplementation, the MP diet (5000 ppm) significantly attenuated the cognitive impairment associated with anti-inflammation, increasing BDNF level and decreasing p-tau (phospho-tau S202) in older B6 mice. We further applied MP dietary supplementation to triple transgenic Alzheimer's disease (3×Tg-AD) mice from 5 to 13 months old. The MP diet exerted neuroprotective, antioxidative, and anti-inflammatory effects and reduced the A*β* deposition and p-tau (S202/S262) levels in the hippocampus of 3×Tg-AD mice, which might further attenuate the deficit in spatial memory retrieval. Thus, these results revealed that the multifunctional properties of MP might offer a promising supplementary diet to attenuate cognitive dysfunction in AD.

## 1. Introduction

Alzheimer's disease (AD) is a major public health crisis in the elderly. The hallmark pathologic features of AD are the accumulation of extracellular senile plaques and intraneuronal neurofibrillary tangles (NFT) in the brain parenchyma [1]. The senile plaques consist of *β*-sheet aggregated amyloid peptides (A*β*) from misprocessing of the amyloid precursor protein (APP), while the NFT are formed by hyperphosphorylation of tau proteins in paired helical filaments [2]. Detection of A*β* oligomers in the cerebrospinal fluid of the postmortem AD brain suggests that soluble A*β* oligomers, but not fibrillar A*β*, are the neurotoxic species in the pathological progression of AD, thus providing a potential target for AD therapy [3]. However, therapeutic strategies targeting A*β* or tau protein within the brain have failed to demonstrate efficacy.

The development of multifunctional therapy is a current trend against multifactorial neurodegenerative disorders such as AD [4]. Polyphenols in natural plants are receiving attention for their multifunction in terms of neuroprotection, antioxidant, and anti-inflammation activities. Evidence suggests that a combination of phytomedicines showed neuroprotective efficacy in preventing neurodegenerative disease [5]. Some natural substances that upregulate the expression of brain-derived neurotrophic factor (BDNF) [6] and anti-inflammation activity [7] could potentially slow the progression of AD.

Mangosteen (*Garcinia mangostana *L.) is a tropical tree native to Southeast Asia that produces a fruit whose pericarp is a rich source of xanthones, which have shown remarkable pharmacological activities [8]. Many studies have suggested that extracts of mangosteen pericarp (MP), such as *α*-mangostin and *γ*-mangostin, have biological functions in terms of anti-inflammation, antioxidation, anticancer, antimicrobial, and neuroprotective activities [9–13]. Recent evidence further suggests that polyphenols in combination induce better protection against cognitive impairment than individual polyphenols [14]. These putative health claims are based on* in vitro *observations and anecdotal reports of the top-selling botanical supplement containing mangosteen fruit in the United States [15]. However, the proposed health benefits of MP have only received very limited attention in terms of cognitive dysfunction in neurodegenerative diseases such as AD. Therefore, in the present study, we evaluated the effects of MP by* in vitro *organotypic hippocampal slice culture (OHSC) and by using* in vivo* B6 and AD mouse systems.

## 2. Materials and Methods

### 2.1. Mice

B6 and 3×Tg-AD (harboring PS1_M146V_, APP_Swe_, and Tau_P30IL_ transgenes) mice were purchased from the National Breeding Center for Laboratory Animals, Taiwan, and the Jackson Laboratory (004807), respectively. B6 male mice (5-week-old) were used to measure the neuroprotection of MP in the organotypic hippocampal slice culture. In addition, the supplement of regular diet and MP diet was administrated in the B6 male (aged 3 weeks and 5 months) and 3×Tg-AD (aged 5 months) mice. The mice were housed in individual-ventilation cages and maintained on a 12 h light/dark cycle at a controlled room temperature and humidity in accordance with standard use protocols and animal welfare regulations. The mice consumed food and water* ad libitum*. All experiments were performed during the light phase between 7 a.m. and 7 p.m. All study protocols were also approved by the Institutional Animal Care and Use Committee of National Taiwan Normal University, Taipei, Taiwan.

### 2.2. Organotypic Hippocampal Slice Culture (OHSC)

OHSCs were conducted in a slightly modified manner from the previous report [16]. Young adult male B6 mice (5-week-old) were anesthetized by Avertin (0.4 g/kg body weight, Sigma, St. Louis, MO, USA) and decapitated. The hippocampi were rapidly dissected in ice-cold artificial cerebrospinal fluid (aCSF) consisting of the following (in mM): NaCl 118, KCl 2.5, MgSO_4_ 3, NaH_2_PO_4_ 1.1, NaHCO_3_ 26, CaCl_2_ 1, and glucose 11 (all reagents from Sigma), bubbled with 95% O_2_/5% CO_2_. Subsequently, coronal slices (350 *μ*m thick) were cut with a vibratome (VT1200S, Leica, Wetzlar, Germany). The sliced hippocampi were transferred onto sterile 0.4 *μ*m porous membrane confetti (Millicell-PCF, Millipore, Ireland) and cultured with medium consisting of 50% MEM, 25% horse serum, 18% HBSS, 4 mM L-glutamine, 12 mM glucose, 4.5 mM NaHCO_3_, 20 mM sucrose, 100 U/mL penicillin, and 100 mg/mL streptomycin (all reagents from Gibco or Sigma) in a humidified 5% CO_2_ atmosphere at 37°C. The medium was changed three times a week. On day* in vitro* (DIV) 5, slices were treated with MP (10 *μ*M) or vehicle (DMSO) for 12 hr. The cell death level of the slices was then examined by PI staining. Furthermore, the BDNF level was examined to evaluate the neuroprotective effect of MP treatment on the hippocampal slices.

### 2.3. PI Uptake Assay in OHSC

PI uptake is an effective assessment of cell damage. At the stated time point, slices were incubated with 5 *μ*g/mL PI (Sigma) in culture medium for 30 min. The stained slices were observed using a fluorescence microscope (Leica, Wetzlar, Germany), and the fluorescent intensity was quantified using AxioVision software (Carl Zeiss, Jena, Germany).

### 2.4. Diets

The male B6 (aged 3 weeks and 5 months) and 3×Tg-AD (aged 5 months) mice were randomly divided into two groups, with 15~17 animals in each group: (i) regular diet (LabDiet 5010, calories provided by protein 27.5%, fat 13.5%, and carbohydrate 59%) and (ii) regular diet with 5000 ppm of MP (Lord Duke Biotechnology Company, containing 35.60 mg/g *α*-mangostin, 0.63 mg/g *β*-mangostin, 1.46 mg/g 3-isomangostin, 1.42 mg/g 8-deoxygartanin, 1.55 mg/g gartanin, and 1.32 mg/g 9-hydroxycalabaxanthone) supplement as in previous studies [15, 17]. Baseline behavioral assessment was conducted in the male 3×Tg-AD mice at 5 months of age. Mice were maintained on MP dietary supplementation for 8 months.

### 2.5. Morris Water Maze Task (MWM)

The Morris water maze task was performed as previously described [18]. In brief, it consists of pretraining, training, testing, and probing. All trials lasted for a maximum of 60 sec. On the day prior to spatial training, all mice underwent pretraining in order to assess their swimming ability and to acclimatize them to the pool. In the three 60 sec pretraining trials, the mouse was released into the water facing the wall of the pool from semirandomly chosen cardinal compass points. After three trials of acclimatization, each mouse was placed on the invisible platform located at the center of the target quadrant and allowed to stay there for 20 sec. The mice were given a 4-day training session consisting of four 60-second training trials (intertrial interval: 20–30 min) per day. The hidden platform was always placed at the same location of the pool (northeast quadrant as the target quadrant) throughout the training period. During each trial, from semirandomly chosen cardinal compass points, the mouse was released into the water facing the pool wall. After climbing onto the platform, the mouse was allowed to rest on it for 20 sec. If the mouse failed to swim to the platform within 60 sec, it would be placed on the platform to stay on it for 20 sec by an experimenter. Twenty-four hours after the last training trial, all mice were given three testing trials to assess the time taken to climb onto the hidden platform. Two and forty-eight hours after the last testing trial, all mice were given two probe trials to evaluate the retrieval of the short- and long-term spatial memory of the platform.

### 2.6. ELISA Analyses

Blood samples were collected from the retroorbital plexus of anesthetized mice and centrifuged at 2,000 ×g for 15 min at 4°C after the MWM (*n* = 3~5 for each group). Sera were collected and stored at −80°C until use. The levels of glutathione (GSH) and IL-6 in the serum were measured using a Glutathione assay kit (Cayman Chemical, MI, USA) and an IL-6 ELISA kit (R&D Systems, MN, USA), respectively. For the BDNF assay, hippocampal slices were homogenized in 8 × volume of lysis buffer containing 50 mM Tris buffer, pH 7.4, 150 mM NaCl, 1% nonidet P-40, 1 mM EDTA, 1 mM phenylmethanesulfonyl fluoride, 1 mM sodium vanadate, 1 mM sodium fluoride, 10 *μ*M aprotinin, 10 *μ*M pepstatin, and 100 *μ*M leupeptin. The protein extracts were diluted with an equal volume of DPBS and further acidified with 1 N HCl to pH 2-3 for 15 min. Subsequently, samples were neutralized with 1 N NaOH. In addition, the concentration of BDNF in the hippocampal slices was measured using a BDNF ELISA kit (Promega, WI, USA). These assays were performed following the manufacturer's instructions.

### 2.7. Western Blot Analysis

Proteins were extracted from the mouse hippocampus (*n* = 3–5 per group). The protein concentration was determined using a bicinchoninic acid (BCA) protein assay kit (Pierce, Rockford, IL, USA). Proteins (25 *μ*g) were separated by SDS-PAGE and transferred to PVDF membranes. The blots were probed with various primary antibodies (Table 1) and secondary antibodies, anti-rabbit and anti-mouse IgG HRP-linked antibody (1 : 10,000; Amersham Pharmacia Biotech, MA, USA). The specific antibody-antigen complex was detected by an enhanced chemiluminescence detection system (Amersham Pharmacia Biotech). The same blot was stripped and reprobed for the housekeeping protein *β*-actin to serve as a loading control. Quantitation was performed using the LAS-4000 chemiluminescence detection system (Fujifilm, Tokyo, Japan), and the target protein density was normalized to the *β*-actin internal control.

### 2.8. Immunostaining

Mice (*n* = 3–5 per group) were anesthetized and transcardially perfused with 0.9% NaCl, followed by 4% paraformaldehyde. Mouse brains were sectioned into 30 *μ*m slices. For immunohistochemical staining, an endogenous peroxidase block was performed for 10 min in 3% H_2_O_2_/PBS. Nonspecific epitopes of free-floating sections were blocked by incubation in 3% normal horse/goat/rabbit serum and 0.1% triton X-100 in PBS. After blocking, sections were incubated in primary antibodies (Table 1) overnight at room temperature, washed with PBS, and incubated with the secondary antibodies (1 : 200 dilution in blocking solution, Vector Laboratories, CA, USA) for 1 h, and then they were incubated in an avidin-biotin complex for 1 h at room temperature. The reaction was developed using a diaminobenzidine (DAB) kit (Vector Laboratories).

For immunofluorescent staining, nonspecific epitopes of free-floating sections were blocked by incubation in 1% BSA in TBST. In addition, sections were hybridized with primary antibodies (Table 1) in blocking solution overnight at 4°C, washed with TBST, and incubated in the secondary antibodies (1 : 500 dilution in blocking solution, Invitrogen AlexaFlour, Eugene, Oregon, USA) for 2 h at 37°C, followed by washing and staining with DAPI (1 : 1000) for another 10 min at room temperature.

All sections were mounted on gelatin-coated slides and cover-slipped for fluorescence/light microscopic observation using a confocal spectral microscope imaging system (Leica TCS SP2, Wetzlar, Germany). The positive staining signal in a specific area was first selected as the standard signal. Then, the number of cells stained positive was counted using digital image analysis software (Image Pro Plus, Media Cybernetics, MD). Pixel counts were taken as the average from three adjacent sections per animal.

### 2.9. Statistical Analysis

Two-way ANOVA was used to analyze “age,” “diet,” and the interactions between them regarding influences on the behavioral and neuropathological measures in the B6 mice. One-way ANOVA was conducted to determine the main effect of diet on the behavioral measures for the 3×Tg-AD mice. ANOVA analyses followed by* post hoc* LSD multiple comparisons when significant were carried out with the most relevant variables from each measurement. In addition, an independent sample *t*-test was used to compare the differences between MP and vehicle in the analysis of neuropathological characterization for OHSC and the 3×Tg-AD mice. Whether the swimming velocity affected the cognitive performance was analyzed by covariate analysis of variance (ANCOVA) with swimming velocity as a covariate. All statistical analyses were performed using SPSS16.0 software. The results are represented as the mean ± SEM. Differences were considered statistically significant when *P* < 0.05.

## 3. Results 

### 3.1. MP Reduced the Cell Death Level Associated with Increasing BDNF Level in Adult Mouse Hippocampal Slices

**Figure 1:**
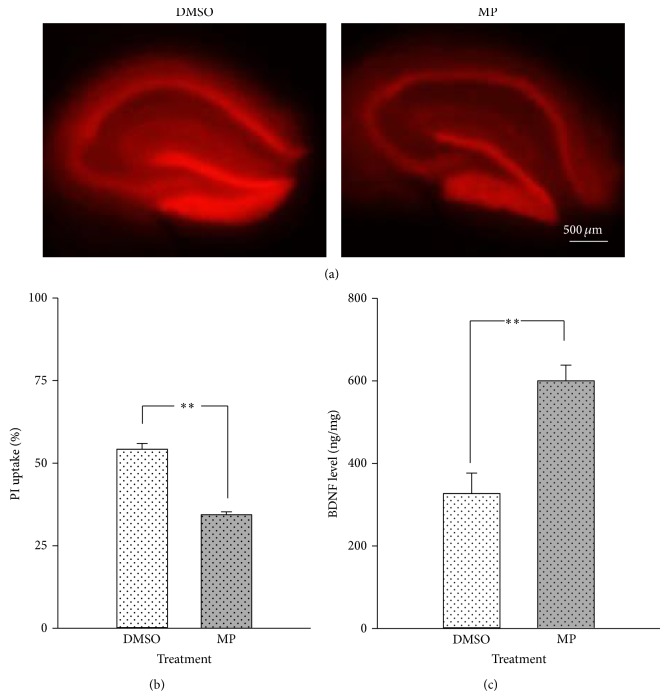
MP induced neuroprotection through increasing BDNF in adult mouse hippocampal slices. (a) The cell death level of hippocampal slices was characterized with PI staining after treatment with MP (10 *μ*M) or DMSO. The scale bar of PI staining is 500 *μ*m. (b) The quantification of PI staining. Treatment with MP significantly decreased the cell death level. (c) The results of BDNF ELISA in the hippocampal slices. Treatment with MP increased the level of BDNF in the hippocampal slices. Data are expressed as means ± SEM, *n* = 9–12 slices/group. ^**^ *P* < 0.01, compared with the DMSO group.

To examine the neuroprotective effect of MP, slices at DIV5 were incubated with 10 *μ*M MP. After 12 h, the hippocampal slices were examined using PI staining and BDNF ELISA. MP treatment significantly attenuated the cell death level (*P* < 0.01; Figures 1(a) and 1(b)) and increased the BDNF level (*P* < 0.05; Figure 1(c)) in the hippocampal slices. These results suggested that MP reduced the cell death level associated with upregulation of the BDNF level in the hippocampal slice cultures.

### 3.2. MP Supplementary Diet Attenuated Cognitive Impairment in Older B6 Mice

**Figure 2:**
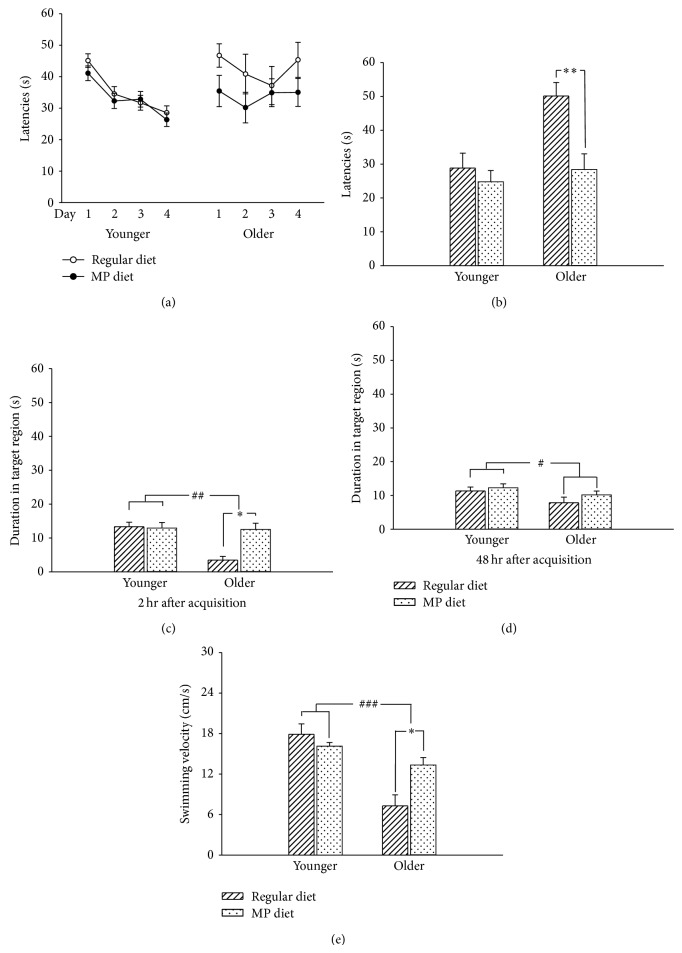
MP diet attenuated the impairments in spatial learning and memory in older B6 mice. (a) The spatial learning ability of B6 mice at different ages and with dietary supplementation. The older B6 mice showed a poor spatial learning ability as compared with the younger mice, and MP dietary supplementation had no influence on the improvement of spatial learning ability. (b) The spatial learning acquisition of B6 mice at different ages and with dietary supplementation. The MP diet increased the spatial learning acquisition in the older B6 mice. (c) The short-term memory retrieval in mice measured 2 h after the last testing trial. The older B6 mice showed impairment in short-term memory retrieval, and MP dietary supplementation attenuated the impairment in the older B6 mice. (d) The long-term memory retrieval in mice measured 48 h after the last testing trial. The older B6 mice showed impairment in long-term memory retrieval, and MP dietary supplementation showed no influence on the impairment. (e) The swimming velocity of the B6 mice at different ages. The decreased swimming velocity of the older B6 mice was rescued by the MP diet. Data are expressed as mean ± SEM, *n* = 15/group. ^#^ *P* < 0.05 and ^###^ *P* < 0.001, comparison between the older and younger groups. ^*^ *P* < 0.05, ^**^ *P* < 0.01, and ^***^ *P* < 0.001, comparison between the regular and MP diet groups.

The effects of age and an MP diet on spatial learning and memory were evaluated by the MWM task. During the training period, the latency to reach the platform in the 4 trials of each training day was averaged and assessed as the spatial learning ability (Figure 2(a)). We found that the older B6 mice that received a regular diet (*P* = 0.61; Figure 2(a)) or an MP diet (*P* = 0.84; Figure 2(a)) did not exhibit decreased latencies onto the platform following training days 1 to 4. However, the younger B6 mice had a good spatial learning ability on a regular diet (*F*_3,59_ = 4.88, *P* < 0.01; Figure 2(a)) and the MP diet (*F*_3,59_ = 3.25, *P* < 0.05; Figure 2(a)). In addition, there were no significant differences in the MP diet or the interaction between age and MP diet on the spatial learning ability (*P* > 0.05; Figure 2(a)).

During the test period, the time spent searching for the platform was assessed as the acquisition of spatial learning. The escape latencies of the older B6 mice were not significantly different from those of the younger B6 mice (*P* = 0.52; Figure 2(b)). In addition, the escape latencies of the mice that were administered MP diet were also not different to those of the mice that were administered a regular diet (*P* = 0.06; Figure 2(b)). There was also no age × MP diet interaction during the testing period (*P* = 0.96; Figure 2(b)).* Post hoc *LSD multiple analyses further showed that the MP diet significantly decreased the escape latencies in the older B6 mice (*P* < 0.01; Figure 2(b)).

Two hours after the last testing trial, the time spent in the target quadrant was assessed as the retrieval of short-term memory. The older B6 mice exhibited a significantly reduced amount of time spent in the target quadrant as compared with the younger B6 mice (*F*_1,42_ = 8.61, *P* < 0.01; Figure 2(c)). The MP diet significantly increased the retrieval of short-term memory as compared with the regular diet (*F*_1,42_ = 6.13, *P* < 0.05; Figure 2(c)). Furthermore, the retrieval of short-term memory was also observed in the interaction between age and MP diet (*F*_1,42_ = 7.32, *P* < 0.01; Figure 2(c)).* Post hoc* analyses further showed that the MP diet significantly increased the time spent in the target quadrant for the older B6 mice (*P* < 0.001; Figure 2(c)). Forty-eight hours after the last testing trial, the time spent in the target quadrant was assessed as the retrieval of long-term memory. We found that the older B6 mice exhibited a significantly reduced retrieval of long-term memory as compared with the younger B6 mice (*F*_1,42_ = 4.21, *P* < 0.05; Figure 2(d)). However, there were no significant differences in the MP diet (*P* = 0.31) and the age × MP diet interaction (*P* = 0.57) for the retrieval of long-term memory.

Furthermore, the swimming velocity was significantly decreased in the older B6 mice as compared with the younger B6 mice (*F*_1,40_ = 37.05, *P* < 0.001; Figure 2(e)). There was no significant difference in the MP diet as compared with the regular diet in swimming velocity (*P* = 0.16; Figure 2(e)). However, there was a significant difference in the interaction between age and MP diet on swimming velocity (*F*_1,40_ = 13.63, *P* < 0.001; Figure 2(e)). According to* post hoc* analysis, the MP diet significantly increased the swimming velocity in the older B6 mice (*P* < 0.05; Figure 2(e)). Therefore, the swimming velocity was significantly correlated with age. These were then entered as covariates in an ANCOVA to examine whether their inclusion was associated with an attenuated cognitive dysfunction on age following MP dietary supplementation. Levene's test of equality of error variances was 0.17. From the results of the two-way ANCOVA analysis, there was no influence to attenuate the cognitive dysfunction on age following MP diet with swimming velocity as a covariant. In addition, after 8-month dietary supplementation with an MP diet or a regular diet, the gain of body weight was increased in both the younger and older B6 mice (data not shown). The blood glucose did not differ among groups separated according to age, MP diet, and age × MP diet (data not shown). Therefore, these results showed that the older B6 mice exhibited impairments in spatial learning and memory. However, the MP supplementary diet attenuated the deficits in spatial learning acquisition and short-term memory retrieval in the older B6 mice.

#### 3.2.1. MP Supplementary Diet Upregulated the BDNF Level and Decreased p-Tau (Phospho-Tau S202) and Inflammatory Response in Older B6 Mice

**Figure 3:**
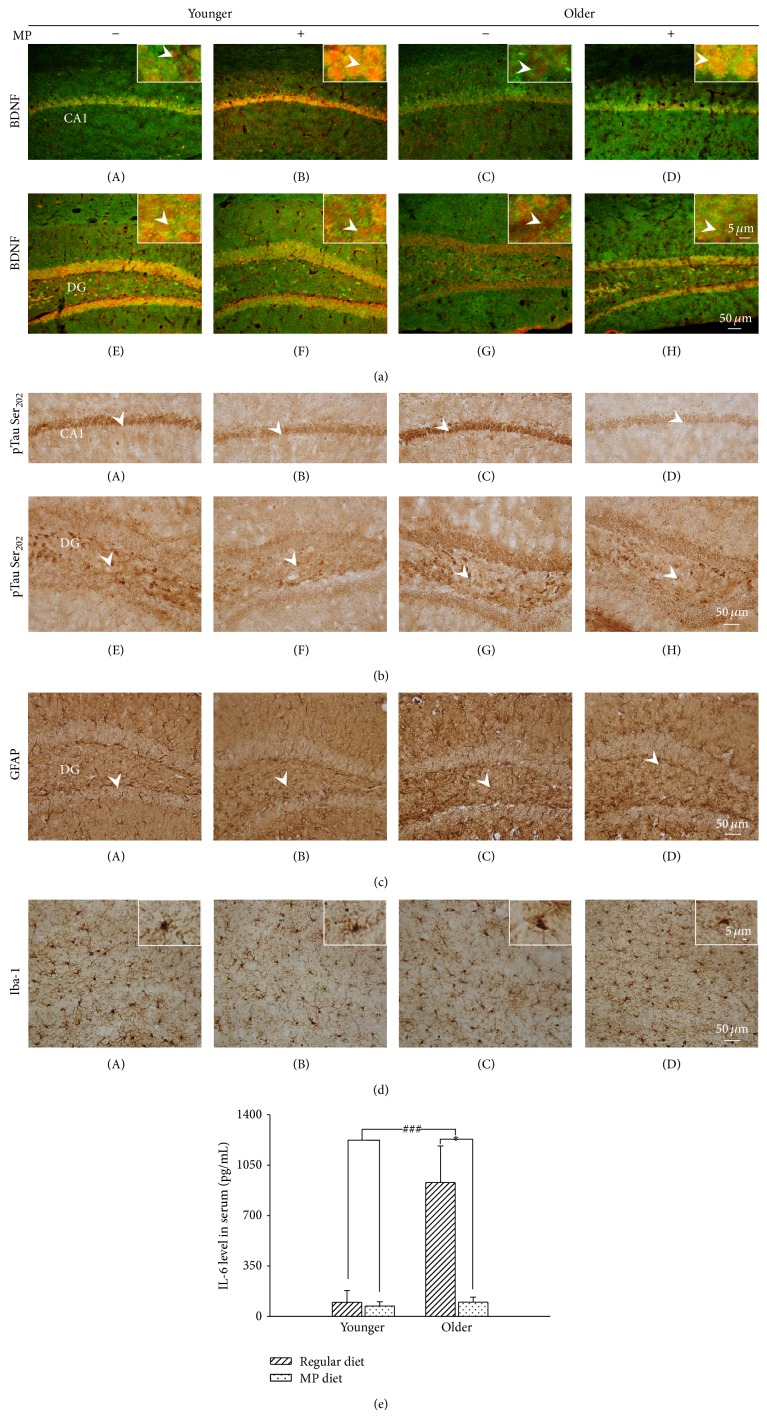
Molecular effects of the MP diet in B6 mice. Representative immunostaining images of BDNF (a), p-tau (S202) (b), activated astrocytes (c), and microglia (d) in the B6 mouse hippocampus. (e) The systemic IL-6 level was determined by ELISA with mouse serum. The IL-6 level was greatly increased in the older B6 mice, which was significantly decreased by the MP diet. All of the deregulations in the older mice were ameliorated after MP treatment. *n* = 3–5/group. Arrowheads indicate positive staining signals. ^##^ *P* < 0.01, comparison between the older and younger groups. ^*^ *P* < 0.05, comparison between the regular and MP diet groups.

Age is a risk factor for the progressive development of AD. In the older B6 mice, the BDNF level was significantly reduced (*F*_1,27_ = 54.08, *P* < 0.001; Figure 3(a) and Table 2) and the p-tau (S202) was significantly increased (*F*_1,25_ = 13.20, *P* < 0.01; Figure 3(b) and Table 2) as compared with younger B6 mice. However, the MP diet significantly increased the level of BDNF (*F*_1,27_ = 92.93, *P* < 0.001; Figure 3(a) and Table 2) and decreased the level of p-tau (S202) (*F*_1,25_ = 31.36, *P* < 0.001; Figure 3(b) and Table 2) in the older B6 mice. For gliosis, there was no significant difference between older and younger B6 mice. MP diet significantly decreased the activated astrocytes (*F*_1,11_ = 10.58, *P* < 0.05; Figure 3(c) and Table 2) and microglia (*F*_1,19_ = 71.62, *P* < 0.001; Figure 3(d) and Table 2). There was also a significant interaction of age × diet in activated microglia (*F*_1,19_ = 10.09, *P* < 0.01; Figure 3(d) and Table 2). When assessing the systematic inflammatory response, the IL-6 level was found to be significantly increased in the older B6 mice as compared with the younger mice (*F*_1,19_ = 17.47, *P* < 0.001; Figure 3(e)), while the MP diet effectively reduced the level (*F*_1,19_ = 17.38, *P* < 0.001; Figure 3(e)). There was also a significant interaction in age × MP diet for the B6 mice (*F*_1,19_ = 15.35, *P* < 0.01; Figure 3(e)). A* post hoc *LSD multiple comparison showed that the MP diet significantly decreased the IL-6 level in the older B6 mice (*P* < 0.05; Figure 3(e)). We also characterized several AD-related targets; however, the molecules involved in the deposition of A*β* (APP, BACE1, A*β*_40_, and A*β*_42_) and tau-related kinases (CDK5, GSK3*β*, JNK, p38, and ERK) were not significantly altered in the older B6 mice (data not shown). These results showed that the older B6 mice exhibited an increased systematic inflammatory response, increased p-tau level (S202), and decreased BDNF level. The MP diet attenuated these impairments in the older B6 mice.

### 3.3. MP Supplementary Diet Attenuated the Deficit in Spatial Memory Retrieval in 3×Tg-AD Mice

**Figure 4:**
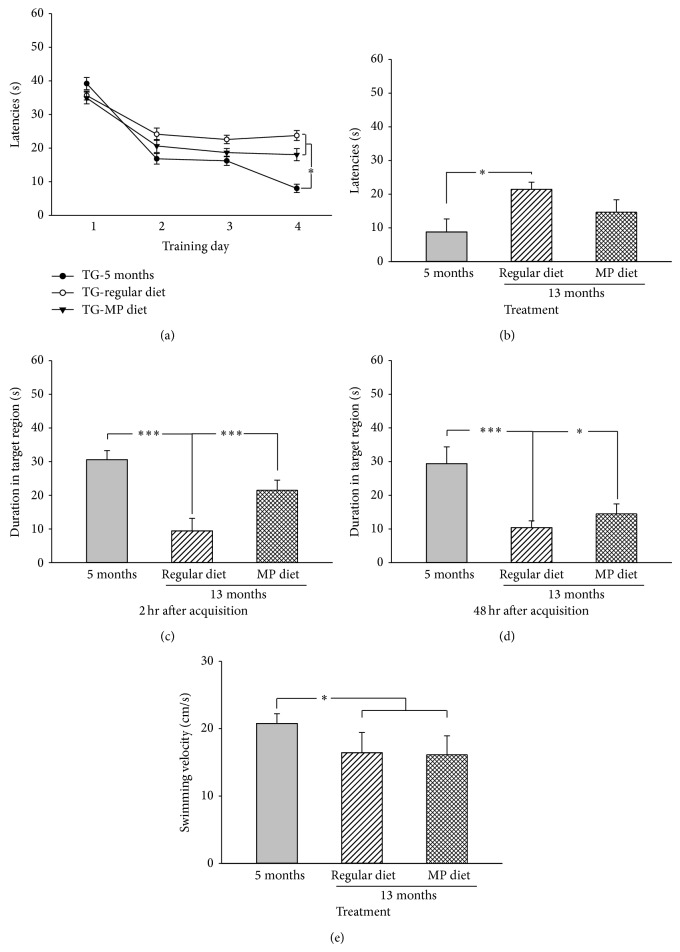
MP diet attenuated the impairment in spatial memory retrieval in 3×Tg-AD mice. (a) The spatial learning ability was measured in the 3×Tg-AD mice from training days 1~4. The 3×Tg-AD mice showed a poor spatial learning ability at 13 months of age, but the MP diet attenuated the impairment in spatial learning ability. (b) The spatial learning acquisition was measured in the 3×Tg-AD mice. The poor spatial learning acquisition of 13-month-old 3×Tg-AD mice could not be rescued by the MP diet. (c) The retrieval of short-term memory in the 3×Tg-AD mice. The reduced time spent in the target quadrant of the 13-month-old 3×Tg-AD mice was significantly increased by the MP diet. (d) The retrieval of long-term memory in the 3×Tg-AD mice. The MP diet significantly increased the time spent in the target quadrant of the 13-month-old 3×Tg-AD mice. (e) The swimming velocity of the 3×Tg-AD mice. The MP diet had no influence on the reduced velocity of the 13-month-old 3×Tg-AD mice. Data are expressed as means ± SEM. *n* = 16-17/group. ^*^ *P* < 0.05, ^**^ *P* < 0.01, and ^***^ *P* < 0.001, compared with the 13-month-old mice treated with a regular diet.

During the training period, we found that a good spatial learning ability was maintained in the 3×Tg-AD mice at 5 months of age (*F*_3,19_ = 30.46, *P* < 0.001; Figure 4(a)), which was declined at 13 months (*F*_3,27_ = 2.51, *P* = 0.08; Figure 4(a)). However, the MP diet restored the spatial learning ability in the 3×Tg-AD mice (*F*_3,23_ = 4.28, *P* < 0.05; Figure 4(a)). For spatial learning acquisition, there was a significant difference between groups (*F*_2,17_ = 4.73, *P* < 0.05; Figure 4(b)). According to* post hoc *LSD multiple comparison, the spatial learning acquisition was significantly declined at 13 months of age as compared with 5 months of age (*P* < 0.05; Figure 4(b)). However, the MP diet had no effect in terms of attenuating the deficit in spatial learning acquisition (*P* = 0.11; Figure 4(b)).

Two hours after the last testing trial, the retrieval of short-term memory was characterized and a significant difference was identified among the 3×Tg-AD mice (*F*_2,17_ = 63.90, *P* < 0.001; Figure 4(c)).* Post hoc* multiple comparison further showed that the retrieval of short-term memory was significantly impaired at 13 months of age as compared with 5 months of age (*P* < 0.001; Figure 4(c)), and the MP diet significantly rescued the deficit in short-term memory of the 13-month-old mice (*P* < 0.001; Figure 4(c)). Forty-eight hours after the last testing trial, the retrieval of long-term memory was assessed, and significant differences were observed between groups (*F*_2,17_ = 49.96, *P* < 0.001; Figure 4(d)). From* post hoc* multiple comparison, we also found that the retrieval of long-term memory was significantly impaired in the 13-month-old mice as compared with the 5-month-old mice (*P* < 0.001; Figure 4(d)), and the MP diet significantly decreased the deficit in long-term memory as compared with the regular diet in the 13-month-old mice (*P* < 0.05; Figure 4(d)).

In addition, the swimming velocity was significantly decreased in the 13-month-old mice as compared with the 5-month-old mice (*P* < 0.05; Figure 4(e)). However, the MP diet did not change the swimming velocity (*P* = 0.83; Figure 4(e)). ANCOVA analysis was performed to determine the diet effect on swimming velocity as a cofactor in the MWM task. We found that swimming velocity had no influence on the MP diet to attenuate the impairment in spatial memory retrieval. Furthermore, both the body weight and blood glucose did not differ between the 3×Tg-AD mice administered the MP diet and the regular diet (data not shown). Therefore, these results indicated that the 3×Tg-AD mice exhibited cognitive dysfunction, and the MP diet attenuated the retrieval impairment of spatial memory at 13 months of age.

#### 3.3.1. MP Supplementary Diet Promoted the Neuroprotection in 3×Tg-AD Mice

**Figure 5:**
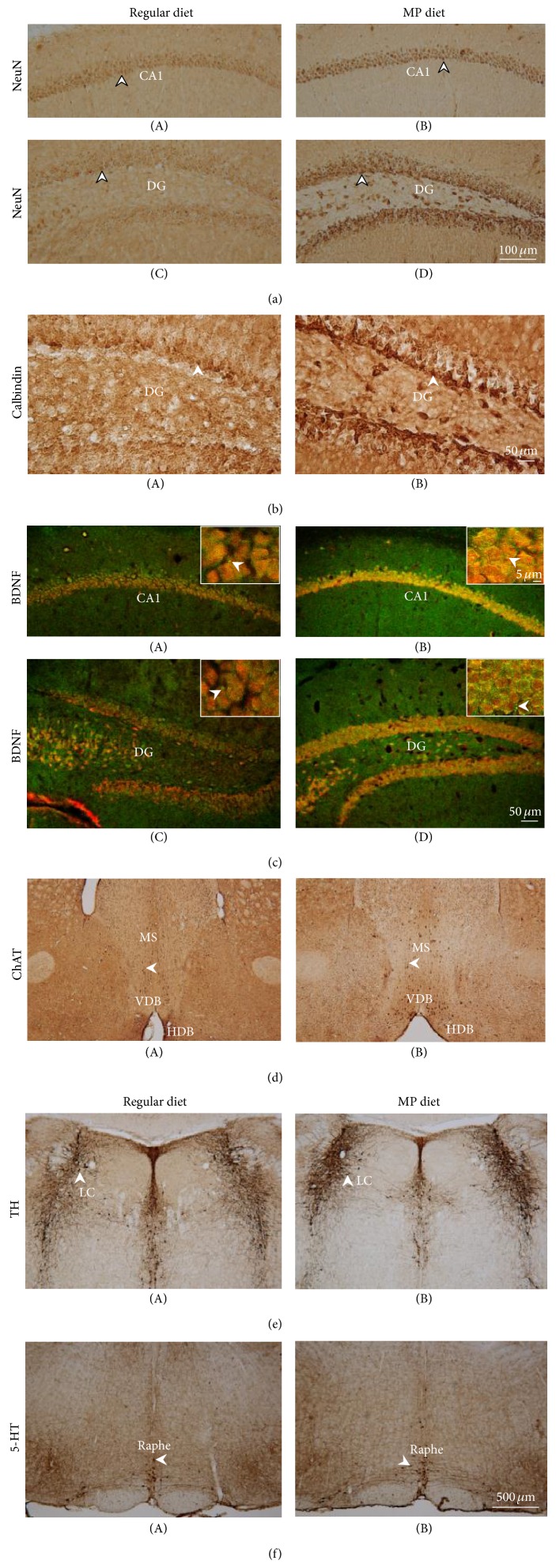
Neuroprotective effects of the MP diet in 3×Tg-AD mice. Immunostaining images of neurons in the hippocampus (a), calbindin levels in the hippocampus (b), BDNF in the hippocampus (c), ChAT in the MS/DB region (d), TH in the LC region (e), and 5-HT in the raphe nucleus (f). Scale bars are 100 *μ*m in panel (a), 50 *μ*m in panels (b) and (c), and 500 *μ*m in panels (d)~(f). Arrowheads indicate positive staining signals. *n* = 3–5/group.

For 3×Tg-AD mice, the MP supplementary diet significantly protected the hippocampal neurons (*P* < 0.01; Figure 5(a) and Table 3) and increased calcium binding protein level in the dentate gyrus (DG) of the hippocampus (*P* < 0.01; Figure 5(b) and Table 3) and an increased BDNF level in the hippocampus (*P* < 0.001; Figure 5(c) and Table 3). In addition, the effects of the MP diet on cholinergic (choline acetyltransferase immunoreactive, ChAT-ir), noradrenergic (tyrosine hydroxylase immunoreactive, TH-ir), and serotonergic (serotonin immunoreactive, 5-HT-ir) neurons were also examined in the mouse brain (Figure 5 and Table 3). MP diet significantly prevented the loss of cholinergic neurons in the medial septum (MS), vertical diagonal band of Broca (VDB), and horizontal diagonal band of Broca (HDB) regions of the 3×Tg-AD mice (*P* < 0.05; Figure 5(d) and Table 3). The MP diet also significantly reduced the loss of noradrenergic neurons in the locus coeruleus (LC) region (*P* < 0.001; Figure 5(e) and Table 3) and serotonergic neurons in the raphe nucleus (*P* < 0.001; Figure 5(f) and Table 3) in the 3×Tg-AD mice. Therefore, these findings showed that the MP diet increased the calcium binding protein and BDNF levels associated with protecting cholinergic, noradrenergic, serotonergic, and hippocampal neurons in the 3×Tg-AD mice. These results suggested that the MP diet might exert neuroprotection via increasing levels of calbindin and BDNF in the 3×Tg-AD mice.

#### 3.3.2. MP Supplementary Diet Reduced the Deposition of A*β* and p-Tau (S202/S262) and Increased the Level of the NR2A/NR2B Ratio in 3×Tg-AD Mice

**Figure 6:**
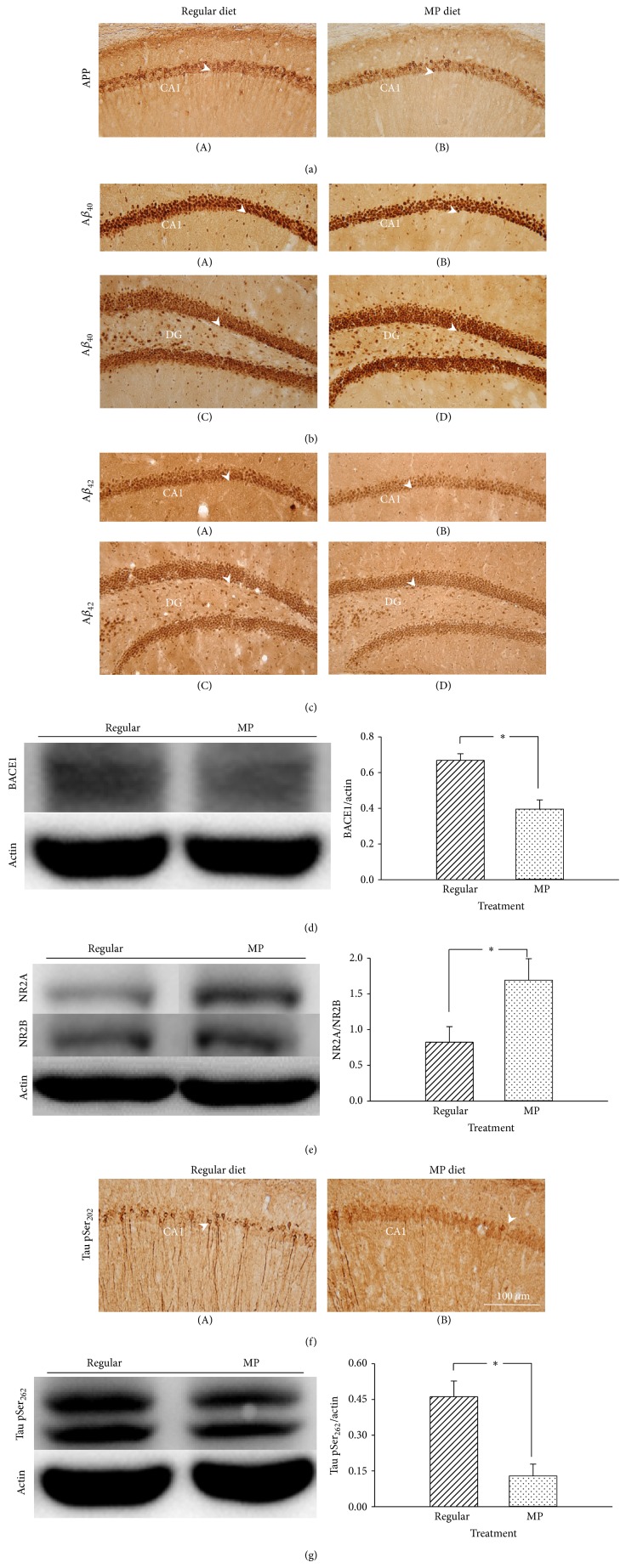
Effects of the MP diet on amyloid deposition, p-tau (S202/S262), and NR2A/NR2B in 3×Tg-AD mice. Immunostaining images of APP (a), A*β*_42_ (b), and A*β*_42_ (c) in the hippocampus of the mice. The levels of BACE1 (d) and NR2A/NR2B ratio (e) in the hippocampus identified by western blot. The MP diet significantly decreased the level of BACE1 and increased the level of the NR2A/NR2B ratio. Immunostaining images of p-tau (S202) (f) in the hippocampus of the mice. (g) The level of p-tau (S262) measured in the hippocampus by western blot. The MP diet greatly decreased the level of p-tau (S262). Scale bar = 100 *μ*m and arrowheads indicate positive staining signals. Data are expressed as means ± SEM. *n* = 3–5/group. ^*^ *P* < 0.05, comparison between the regular and MP diet groups.

There was no significant difference between regular and MP diet in the levels of APP (*P* = 0.54; Figure 6(a) and Table 3) and A*β*_40_ (*P* = 0.29; Figure 6(b) and Table 3). However, MP diet significantly decreased the levels of A*β*_42_ (*P* < 0.05; Figure 6(c) and Table 3) and BACE1 (*P* < 0.05; Figure 6(d)) in the hippocampus as compared with regular diet. We further found that the level of the NR2A/NR2B ratio in the hippocampus was significantly increased in the mice administered the MP diet as compared with a regular diet (*P* < 0.05; Figure 6(e)). In addition, the phosphorylated levels of p-tau (S202) (*P* < 0.001; Figure 6(f) and Table 3) and p-tau (S262) (*P* < 0.05; Figure 6(g)) were also reduced after administration of the MP diet. However, there were no significant differences identified in the other related molecules, including the total A*β* level, total tau level, inactive GSK3*β* (pS9), CDK5, and several signaling kinases (ERK, JNK, and Akt) (data not shown). Therefore, these results indicated that the MP diet largely reduced the amyloidal deposition and p-tau level (S202/S262) and increased the level of the NR2A/2B ratio in the hippocampus of the 3×Tg-AD mice.

#### 3.3.3. MP Supplementary Diet Decreased Oxidative Stress and Inflammatory Responses in 3×Tg-AD Mice

**Figure 7:**
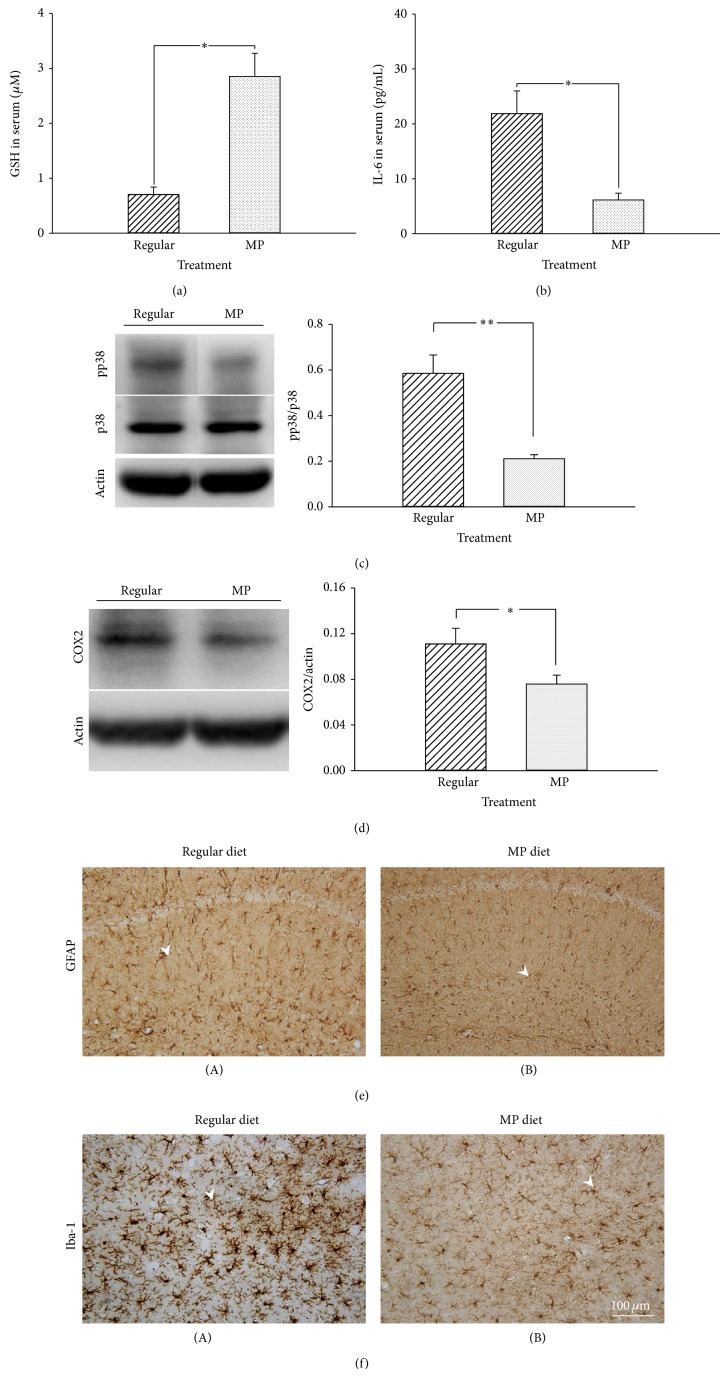
The antioxidative and anti-inflammatory activities of MP in 3×Tg-AD mice. (a) The systemic GSH level was determined by ELISA with mouse serum. MP diet significantly increased the GSH level in the mouse serum. (b) The systemic IL-6 level was determined by ELISA with mouse serum. MP diet significantly decreased the IL-6 level in the mouse serum. (c) The level of pp38 was measured in the hippocampus of the mice by western blot. MP diet significantly decreased the level of pp38. (d) The level of COX2 was measured in the hippocampus of the mice by western blot. MP diet significantly decreased the level of COX2. Immunostaining images of activated astrocytes (e) and microglia (f) in the hippocampus. Scale bar = 100 *μ*m and arrowheads indicate positive staining signals. Data are expressed as means ± SEM. *n* = 3–5/group. ^*^ *P* < 0.05 and ^**^ *P* < 0.01, comparison between the regular and MP diet groups.

We also found that the MP diet significantly increased the serum GSH and decreased the serum IL-6 concentration as compared with the regular diet (*P* < 0.05; Figures 7(a) and 7(b)). Moreover, a series of inflammatory-related signaling pathways involved in pathogenesis of AD animal models and patients [19–21] were also characterized in this study. Among these pathways, we further found that both the levels of phosphorylated p38 MAPK (*P* < 0.01; Figure 7(c)) and COX2 (*P* < 0.05; Figure 7(d)) were decreased in the 3×Tg-AD mice administered an MP diet. From the immunostaining analysis of the mouse hippocampus, we observed that the MP diet significantly decreased the activation of astrocytes (GFAP positive staining) and microglia (Iba1 positive staining with round or amoeboid cells) as compared with the regular diet (*P* < 0.05 and *P* < 0.001, resp.; Figures 7(e)-7(f) and Table 3). These results showed that the MP diet exerted anti-inflammatory and antioxidative activities in the 3×Tg-AD mice.

## 4. Discussion

In this study, the neuroprotective property of MP treatment was first evaluated using an OHSC platform. Furthermore, the effects and molecular mechanisms of the long-term MP supplementary diet were elucidated in both B6 and 3×Tg-AD male mice. We demonstrated that (1) MP treatment exhibited neuroprotective activity via increasing the BDNF level in hippocampal slices; (2) the MP diet attenuated the cognitive impairment associated with an increasing BDNF level, increased anti-inflammation, and decreased p-tau level (S202) in older B6 male mice; and (3) the MP diet also attenuated the deficit in spatial memory retrieval associated with increases in antioxidation, anti-inflammation, the NR2A/NR2B ratio, neurotransmitter neurons, hippocampal neurons, calcium binding protein, and BDNF level and decreased A*β*_42_, BACE1, activated glia cells, and p-tau (S202/S262) in 3×Tg-AD male mice. This was the first study to demonstrate the multifunctional properties of MP in terms of attenuating the cognitive dysfunction of AD.

At first, the polyphenolic xanthone-enriched MP significantly reduced the cell death level associated with an increasing BDNF level in hippocampal slices. This result is consistent with previous evidence showing that the natural product possesses a potential neuroprotective activity for the treatment of neurodegenerative diseases [22, 23]. In an* in vivo *study, we found that the older B6 mice had cognitive dysfunction associated with increasing systematic IL-6 and p-tau levels (S202) and a decreasing BDNF level, and MP dietary supplementation attenuated these impairments significantly. Accumulating evidence indicates that BDNF is critical for the survival and guidance of neurons to influence the long-term potentiation, neuroplasticity, learning, and memory [24, 25]. Evidence suggests that low BDNF is correlated with high IL-6 in the cognitive dysfunction of multiple sclerosis patients [26]. Recent study has also shown that a chronic inflammatory state resulted from increased secretion of proinflammatory cytokines and mediators in the elderly [27]. Previous study has shown that a systemic immune challenge in wild-type mice might play an important role in inducing tau protein phosphorylation to develop an AD-like neuropathology during the course of aging [28]. Recent evidence further indicated that inflammatory mediators such as IL-6 could modulate tau phosphorylation independent of the A*β* levels in a mouse model [29]. Therefore, these findings suggest that the cognitive dysfunction shown in the older B6 mice is associated with p-tau (S202), inflammation, and reduced BDNF. MP dietary supplementation could effectively attenuate these impairments in mice.

In this study, we observed that 3×Tg-AD mice showed cognitive dysfunction at 13 months of age as compared with 5 months of age. The MP diet alleviated the cognitive impairment associated with increases in serum GSH, BDNF, the NR2A/NR2B ratio, calbindin, neurotransmitter neurons, and hippocampal neurons and decreases in serum IL-6 level, activated glia, pp38, COX2, p-tau (S202/S262), A*β*_42_, and BACE1 in the hippocampus of 3×Tg-AD mice. However, no significant alterations were observed in CDK5, GSK3*β*, JNK, ERK, total tau, and total A*β* in the hippocampus of the 3×Tg-AD mice (data not shown). Evidence has also revealed that the impairment of social recognition in 3×Tg-AD mice was not associated with increasing total tau and A*β* deposition [30]. MP dietary supplementation attenuated the cognitive dysfunction associated with an increasing BDNF level and decreasing inflammatory-related signals (IL-6, pp38, COX2, and activated glia cells) and p-tau (S202/S262). The same results were also observed in the older B6 mice, except for pp38, COX2, and p-tau (S262). Previous study has shown that both pp38 and COX-2 are upregulated in AD transgenic mice [31].

In addition, MP dietary supplementation also decreased oxidative stress and the deposition of A*β*_42_ associated with a reducing BACE1 level. Evidence also shows that BACE1 inhibitor impacted amyloid deposition [32]. Previous study further showed that the methanol extract of MP attenuates A*β*_42_-induced ROS in SK-N-SH cells [33]. GSH is the most abundant intracellular antioxidant that protects cells against oxidative damage caused by ROS [34, 35]. Therefore, these results revealed that the MP diet attenuated the cognitive dysfunction associated with antioxidative, anti-inflammatory, and neurotrophic activity through decreasing the deposition of A*β*_42_ and tau protein phosphorylation in the AD mice.

Impaired synaptic function has been linked with the AD pathological process [36]. NMDARs are known to maintain the synaptic plasticity and contribute to memory formation [37]. The bioactivity of NMDARs regulates synaptic function and neurotransmission to sustain normal long-term potential (LTP) and memory formation [38, 39]. LTP requires activation of the NR2A subunit but not the NR2B subunit [40]. Evidence also suggests that a synaptic plasticity alteration was associated with a decrease in the NR2A/NR2B ratio in both a neurotoxic and transgenic model of Parkinson's disease [41]. Previous study further suggested that a high NR2A/NR2B ratio would be required for LTP induction [42]. In this study, the MP diet restored the spatial memory retrieval associated with increasing levels of the NR2A/NR2B ratio, cholinergic neurons in MS/DB, serotonergic neurons in the Raphe nucleus, noradrenergic neurons in the LC region, and calcium-binding protein calbindin D28K in the DG subregion of the hippocampus of 3×Tg-AD mice. Our previous studies also showed that the NR2A/NR2B ratio, calbindin, and neurons (cholinergic, serotonergic, and noradrenergic) involved in neurotransmission play pathogenic roles in memory loss in AD [43]. Evidence also shows that symptomatic drug treatment for AD might be beneficially directed toward ameliorating multiple neurotransmitter deficiencies [44]. Recent evidence further indicated that calbindin depletion might be an important contributor to the pathogenesis of AD [45]. Therefore, these results showed that the MP diet attenuated the spatial memory impairment associated with the protection of cognitive-related signals, the NR2A/NR2B ratio, the neurotransmitter neurons, and calcium-binding protein in 3×Tg-AD mice. In conclusion, our results demonstrated that the therapeutic strategy of MP dietary supplementation attenuated cognitive dysfunction via multifunctional properties. Therefore, the multifunctional strategy might be a potential therapy against multifactor-mediated AD.

## Figures and Tables

**Table 1:** List of the primary antibodies used in this study.

Antibodies	Species	Supplier	WB dilution	IHC dilution	Epitope specificity
Amyloid beta antibodies					
A*β*_1–40_	Rabbit	Invitrogen	—	1 : 2,000	Amyloid beta
A*β*_1–42_	Rabbit	Invitrogen	—	1 : 500	Amyloid beta
APP	Rabbit	Sigma-Aldrich	—	1 : 500	Amyloid precursor protein
BACE	Rabbit	Cell Signaling	1 : 1,000	—	Beta secretase
Neurotransmission antibodies					
5-HT	Rat	Millipore	—	1 : 100	Serotonergic neurons
Calbindin	Mouse	Sigma-Aldrich	—	1 : 1,000	Calcium binding protein
ChAT	Rabbit	Millipore	—	1 : 500	Cholinergic neurons
NeuN	Mouse	Millipore	—	1 : 1,000 1 : 500 (IF)	Neuronal cells
NR2A	Rabbit	Millipore	1 : 1,000	—	NMDA receptor 2A
NR2B	Rabbit	Millipore	1 : 1,000	—	NMDA receptor 2B
TH	Rabbit	Millipore	—	1 : 1,000	Noradrenergic neurons
Inflammation antibodies					
COX2	Rabbit	Millipore	1 : 1,000	—	Prostaglandin synthase-2
GFAP	Mouse	Millipore	—	1 : 1,000 1 : 500 (IF)	Astrocytes
Iba-1	Rabbit	Wako	—	1 : 1,000	Microglia
Signaling antibodies					
Akt 1/2	Rabbit	Cell Signaling	1 : 1,000	—	Total Akt
pAkt 1/2	Rabbit	Cell Signaling	1 : 1,000	—	Akt phosphorylated at Thr^450^
CDK5	Mouse	Millipore	1 : 1,000	—	Cyclin-dependent kinase-5
Erk 1/2	Rabbit	Cell Signaling	1 : 1,000	—	Total Erk 1/2
pErk 1/2	Rabbit	Cell Signaling	1 : 1,000	—	Erk 1/2 phosphorylated at Thr^202^/Tyr^204^
GSK3*α*	Rabbit	Cell Signaling	1 : 1,000	—	Total GSK3*α*
pGSK3*α*	Rabbit	Cell Signaling	1 : 1,000	—	GSK3*α* phosphorylated at Ser^21^
GSK3*β*	Rabbit	Epitomics	1 : 1,000	—	Total GSK3*β*
pGSK3*β*	Rabbit	Epitomics	1 : 1,000	—	GSK3*β* phosphorylated at Ser^9^
JNK	Rabbit	Cell Signaling	1 : 1,000	—	Total JNK
pJNK	Rabbit	Cell Signaling	1 : 1,000	—	JNK phosphorylated at Thr^183^/Tyr^185^
p38	Rabbit	Cell Signaling	1 : 1,000	—	Total p38
pp38	Rabbit	Cell Signaling	1 : 1,000	—	p38 phosphorylated at Thr^180^/Tyr^182^
Tau antibodies					
pTau	Rabbit	AnaSpec	—	1 : 1,000	Tau hyperphosphorylated at Ser^202^
pTau	Rabbit	Millipore	1 : 1,000	—	Tau hyperphosphorylated at Ser^262^
Tau1	Mouse	Millipore	1 : 1,000	—	Tau unphosphorylation form
Other antibodies					
*β*-Actin	Mouse	Millipore	1 : 2,000	—	*β*-Actin
BDNF	Rabbit	Millipore	—	1 : 500 (IF)	Brain-derived neurotrophic factor

WB: Western blot; IHC: immunohistochemistry; IF: immunofluorescence.

**Table 2:** The results of immunostaining in C57BL/6 mice after dietary supplementation.

Target	Younger		Older	
	Regular diet	MP diet	Regular diet	MP diet
BDNF	31 ± 0.94	45 ± 1.60^∗∗∗b^	21 ± 1.81^∗∗∗a^	34 ± 1.23^∗∗∗b^
Tau pSer202	206 ± 8.21	116 ± 17.48^∗∗b^	281 ± 22.55^∗a^	171 ± 7.73^∗∗∗b^
GFAP	39 ± 2.87	35 ± 0.52	46 ± 2.53	36 ± 2.36^∗b^
Iba-1	44 ± 1.16	35 ± 0.72^∗∗∗b^	43 ± 0.50	39 ± 0.51^∗∗∗b^

Each value represents the mean ± SEM (*n* = 3–5 for each group).

^a^Older mice compared with younger mice.

^b^Regular diet compared with MP diet group.

^*^ *P* < 0.05; ^**^ *P* < 0.01; ^***^ *P* < 0.001.

**Table 3:** The results of immunostaining in 3×Tg-AD mice after dietary supplementation.

Target	Regular diet	MP diet
NeuN	716 ± 28.69	1037 ± 78.30^***^
Calbindin	20 ± 3.71	91 ± 9.91^**^
BDNF	19 ± 1.01	27 ± 0.67^***^
ChAT	42 ± 5.00	62 ± 5.80^*^
TH	57 ± 4.66	111 ± 5.64^***^
5-HT	36 ± 2.83	67 ± 5.41^***^
APP	165 ± 4.07	159 ± 6.62
A*β*_40_	613 ± 29.39	656 ± 20.08
A*β*_42_	685 ± 14.72	604 ± 23.01^#^
Tau pSer202	37 ± 2.22	19 ± 2.44^###^

Each value represents the mean ± SEM (*n* = 3–5 for each group).

All values were compared with the regular diet group.

^*^Increased (*P* < 0.05); ^**^increased (*P* < 0.01); ^***^increased (*P* < 0.001).

^#^Decreased (*P* < 0.05); ^##^decreased (*P* < 0.01); ^###^decreased (*P* < 0.001).
